# Third Nerve Palsy: A Case of Bilateral Eyelid Ptosis, Normal Pupils, and Vertical Diplopia With Multiple Intracranial Lesions

**DOI:** 10.7759/cureus.93277

**Published:** 2025-09-26

**Authors:** Roxana M Dragomir, Octavio Carranza-Rentería, Marc A Swerdloff, Matthew Kay

**Affiliations:** 1 Neurology, Florida Atlantic University Charles E. Schmidt College of Medicine, Boca Raton, USA; 2 Neurology, Baptist Health Medical Group North, Boynton, USA; 3 Marcus Neuroscience Institute, Boca Raton Regional Hospital, Boca Raton, USA; 4 Neuro-Ophthalmology, Florida Atlantic University Charles E. Schmidt College of Medicine, West Palm Beach, USA

**Keywords:** case report, diplopia, midbrain lesion, nuclear third nerve, ptosis

## Abstract

Bilateral eyelid ptosis presents a rare and diagnostically challenging clinical scenario, particularly when accompanied by multiple intracranial lesions. We present the case of a 68-year-old woman with bilateral ptosis, vertical diplopia, and progressive confusion. Imaging revealed a dorsal midbrain lesion and bilateral frontal lobe masses. Clinical examination ruled out common causes of ptosis, such as myasthenia gravis and muscular dystrophies. The patient’s constellation of symptoms, bilateral ptosis without pupillary involvement and vertical diplopia, localized the lesion to the midbrain rather than the cortex. This case highlights the importance of integrating clinical examination with neuroimaging to refine localization in challenging presentations of cranial nerve pathology.

## Introduction

Bilateral ptosis is a rare and diagnostically complex condition with a broad differential. While lesions of the central caudal nucleus (CCN) in the midbrain may present with symmetrical bilateral ptosis due to dysfunction of the levator palpebrae superioris muscles, asymmetric nuclear third nerve lesions have also been described [1,2]. Pupillary involvement is not a reliable indicator for lesion localization, as both nuclear and infranuclear third nerve lesions may affect or spare the pupil [3]. Bilateral ptosis may result from frontal cortical or rostral midbrain lesions. If present, ocular motility deficits strongly support midbrain involvement [4]. Here, we present a rare case of bilateral eyelid ptosis with preserved pupillary function and vertical diplopia, associated with multiple intracranial lesions, highlighting the importance of neuroimaging in lesion localization.

## Case presentation

**Figure 1:**
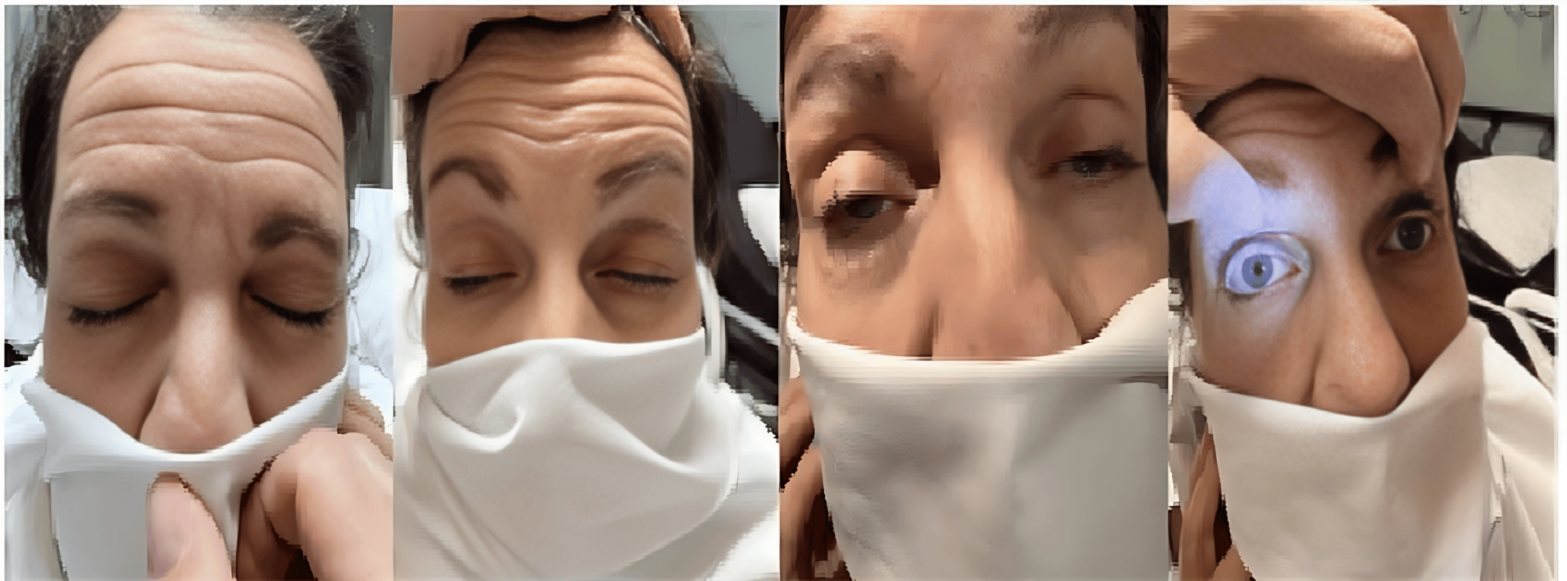
Profound bilateral ptosis and normal pupillary response. Clinical photograph demonstrating profound bilateral ptosis with preserved pupillary light and accommodation responses. No anisocoria was noted.

A left-handed 68-year-old woman was referred to our hospital for one month of intermittent vertical diplopia, bilateral lid ptosis, progressive confusion, and a dull, unremitting holocranial headache. An outpatient noncontrast MRI of the brain reportedly revealed bilateral frontal lobe masses. Initial examination showed she was awake and alert without aphasia or neglect. She had bilateral ptosis without blepharospasm. Figure 1 shows profound bilateral ptosis.

An ice pack over the eye for two minutes did not improve the ptosis. Visual fields were intact to finger confrontation with lids manually opened. Visual acuity was 20/20 in both eyes. Her pupils were symmetric and reactive to light and accommodation. Extraocular movements were full in all directions of gaze without nystagmus. The cover-uncover test showed no skew deviation, although prism measurements were not conducted. After prolonged speaking, neither dysarthria nor dysphonia appeared. Fatigable muscle weakness was not precipitated by repetitive contractions of the proximal or distal muscles of her extremities. Heel-to-shin and finger-to-nose testing were normal without dysmetria. Facial and body sensation was normal to light touch, vibration, and pinprick. Bilateral biceps, triceps, patellar, and ankle reflexes were normal. Extensor plantar responses were absent bilaterally. Her gait was limited by profound bilateral eye ptosis. The palmomental reflex and snout reflexes were absent.

**Figure 2:**
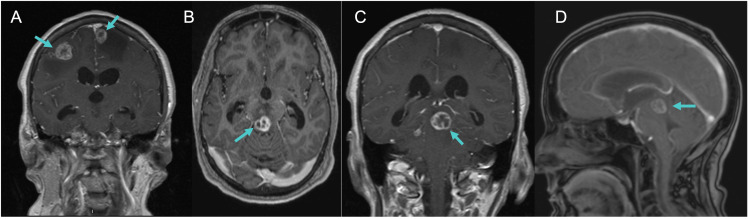
Contrasted MRI brain demonstrating bilateral frontal lesions and a lesion in the dorsal midbrain. (A) Coronal view of T1 post-gadolinium sequence. Blue arrows point to two lesions in the frontal lobe. (B) Axial view T1 post-gadolinium sequence at the level of the midbrain. Blue arrow points to contrast enhancing lesion near the tectum. (C) Coronal view T1 post-gadolinium. Blue arrow points to a contrast-enhancing lesion in the midbrain. (D) Sagital view T1 post-gadolinium. Blue arrow points to an enhancing lesion in the midbrain.

Repeat contrasted brain MRI performed during the patient’s hospitalization showed a 1.5 cm ring-enhancing lesion in the dorsal midbrain near the tectum, compressing the cerebral aqueduct, causing obstructive hydrocephalus, and two ring-enhancing lesions in the frontal lobes with surrounding vasogenic edema (Figure 2).

The patient underwent CT body imaging due to the suspicion of metastatic cancer to the brain. There was no evidence of a primary tumor of the chest, abdomen, or pelvis. She was prescribed dexamethasone to lessen vasogenic brain edema. After 24 hours of treatment, she could partially open the right eyelid without supplemental frontalis muscle contraction, but her left eyelid remained ptotic.

Biopsy showed a mixture of necrosis and hemorrhagic material initially. The final report was negative for metastatic carcinoma, glioma, lymphoma, vasculitis, infarction, abscess, or encephalitis. Immunohistochemistry searching for cancer markers was negative. After treatment with dexamethasone for vasogenic brain edema, the patient had asymmetric improvement of her ptosis, more so on the right. A second biopsy at another institution was again negative for cancer. She became progressively more confused, deteriorated shortly after, and was discharged to hospice.

## Discussion

The differential diagnosis of bilateral lid ptosis includes congenital ptosis, mechanical ptosis from fullness of the upper eyelids (secondary to infection, inflammation, or fat accumulation), myasthenia gravis, Lambert-Eaton syndrome, metabolic and inherited myopathies, recent exposure to botulinum toxin, third cranial nerve (CN3) dysfunction, and apraxia of eyelid opening [5-10].

Our patient reported no pre-existing lid ptosis, exposure to botulinum toxin, ocular infection, or ocular surgery. There was no anisocoria to suggest Horner’s syndrome. There was no diurnal variation of ptosis or improvement with application of ice packs to the lids to suggest neuromuscular transmission failure. Lid inspection was normal without signs of infection, edema, or dermatochalasis. There was no muscle weakness, dysphagia, or dysarthria to suggest chronic progressive external ophthalmoplegia, oculopharyngeal muscular dystrophy, myotonic dystrophy, myasthenia gravis, or Lambert-Eaton syndrome [11,12].

Ptosis results from incomplete opening of the eyelid through musculoskeletal or neurologic mechanisms. Neurogenic ptosis is an important neurological sign and should always be investigated [12]. Our differential diagnosis included a true neurogenic ptosis from either a bilateral supranuclear CN3 palsy, i.e., apraxia of eyelid opening from failure to disinhibit the levator, versus a nuclear CN3.

Apraxia of eyelid opening has been described with cerebral lesions in the non-dominant hemisphere, basal ganglia, rostral brainstem, or bilateral mesial frontal lobe lesions. Apraxia of eyelid opening is also seen in movement disorders such as Parkinson’s disease, corticobasilar degeneration, multiple system atrophy, and progressive supranuclear palsy, or as a side effect of medications (lithium intoxication or neuroleptic medications) [13,14]. Our patient had none of these latter conditions. She had bilateral frontal lobe lesions that could conceivably result in the development of bilateral ptosis; however, they would not account for vertical diplopia. Accordingly, the midbrain lesion, located with reference to the nucleus of the oculomotor nerve, was most likely the etiology for the patient’s clinical presentation of bilateral ptosis and diplopia.

The most frequent oculomotor nerve injury occurs at the fascicles of the CN3 after emerging from the nucleus. When affected, a patient presents with ophthalmoplegia, pupillary abnormalities, and unilateral ptosis ipsilateral to the lesion. Our patient did not have papillary abnormalities. Bilateral ptosis is infrequent and occurs specifically with nuclear lesions of the CCN. Lesions in this midline structure present with bilateral ptosis, also known as midbrain ptosis. Typically, this type of ptosis will present with other signs of midbrain injury [15].

Eyelid opening and closure are under the control of the central and autonomic nervous system structures [16]. Voluntary opening of the eyelid occurs by contraction of the levator palpebrae superioris. This muscle is innervated by the oculomotor nerve. Muller’s muscle in the superior border of the upper tarsus is an accessory eyelid retractor and assists in the elevation of the upper lid. This muscle is innervated by fibers from the ipsilateral cervical ganglion of the sympathetic chain. The centrally innervated muscles minimally contribute to eyelid appearance and elevation.

Neurogenic ptosis can be characterized with respect to the nucleus of CN3. The oculomotor nucleus is a midbrain midline structure ventral to the periaqueductal gray matter at the level of the superior colliculus. It is a collection of neurons from which the fibers of the third cranial nerve originate. Lesions affecting the oculomotor nerve may occur at the nucleus of CN3 (nuclear third), inferior to the nucleus (infranuclear), or above the nucleus (supranuclear) [16-18].

The CN3 nucleus can be subdivided into several subnuclei. Each subnucleus sends fibers to specific muscles innervated by the oculomotor nerve. The CCN innervates the bilateral levator palpebrae superioris. The other subnuclei are bilateral paired structures. The Edinger-Westphal nucleus supplies the ipsilateral pupillary sphincter constrictor muscles, joining up with the motor fibers at the infranuclear level. All axons from these subnuclei transverse the red nucleus, exit the midbrain at the interpeduncular fossa, and emerge in the subarachnoid space as the oculomotor nerve.

Supranuclear ptosis, also called cortical ptosis, can be unilateral or bilateral. When unilateral, it presents with ptosis in the eye contralateral to the lesion. Cortical ptosis is caused by multiple etiologies affecting the cortex. Apraxia of eyelid opening is a supranuclear inability to open the eyelids from simultaneous inhibition of bilateral levator palpebrae superioris and/or prolonged action of the orbicularis oculi [18]. The term was first coined in 1965 by Goldstein and Cogan and described as a nonparalytic motor abnormality characterized by difficulty initiating lid elevation [19]. Aphasia must not be present to prevent understanding of the command to open the eyes. There must be an intact effector mechanism, viz., no lesion of the cranial nerves or impairment of the oculomotor muscles.

## Conclusions

The patient’s constellation of findings, i.e., bilateral eyelid ptosis without pupillary involvement, vertical diplopia, and preserved extraocular movements, strongly suggested a nuclear third cranial nerve lesion, most likely involving the CCN of the midbrain. While bilateral frontal lobe lesions may contribute to cognitive dysfunction and apraxia of eyelid opening, they do not account for the ocular motor deficits observed. The midbrain lesion, therefore, represents the most plausible anatomical correlate for the patient’s ophthalmologic presentation. This case highlights the critical role of neuroanatomical localization in the diagnostic evaluation of bilateral ptosis and vertical diplopia, particularly in the context of multiple intracranial lesions.
